# Molecular Pathology of Poorly Differentiated and Anaplastic Thyroid Cancer: What Do Pathologists Need to Know?

**DOI:** 10.1007/s12022-021-09665-2

**Published:** 2021-02-04

**Authors:** Marco Volante, Alfred K. Lam, Mauro Papotti, Giovanni Tallini

**Affiliations:** 1 Department of Oncology, University of Turin, Turin, Italy; 2 School of Medicine, Griffith University, Gold Coast, Australia; 3 Department of Experimental, Diagnostic and Specialty Medicine, University of Bologna School of Medicine, Bologna, Italy

**Keywords:** Poorly differentiated, Anaplastic, Carcinoma, Thyroid, Molecular, Progression

## Abstract

The molecular characterization of poorly and anaplastic thyroid carcinomas has been greatly improved in the last years following the advent of high throughput technologies. However, with special reference to genomic data, the prevalence of reported alterations is partly affected by classification criteria. The impact of molecular pathology in these tumors is multifaceted and bears diagnostic, prognostic, and predictive implications although its use in the clinical practice is not completely assessed. Genomic profiling data claim that genetic alterations in poorly differentiated and anaplastic thyroid carcinomas include “Early” and “Late” molecular events, which are consistent with a multi-step model of progression. “Early” driver events are mostly *RAS* and *BRAF* mutations, whereas “Late” changes include above all *TP53* and *TERT* promoter mutations, as well as dysregulation of gene involved in the cell cycle, chromatin remodeling, histone modifications, and DNA mismatch repair. Gene fusions are rare but represent relevant therapeutic targets. Epigenetic modifications are also playing a relevant role in poorly differentiated and anaplastic thyroid carcinomas, with altered regulation of either genes by methylation/deacetylation or non-coding RNAs. The biological effects of epigenetic modifications are not fully elucidated but interfere with a wide spectrum of cellular functions. From a clinical standpoint, the combination of genomic and epigenetic data shows that several molecular alterations affect druggable cellular pathways in poorly differentiated and anaplastic thyroid carcinomas, although the clinical impact of molecular typing of these tumors in terms of predictive biomarker testing is still under exploration.

## Introduction

Histological classification of thyroid cancers deriving from follicular cells is designed to keep separate the well-differentiated thyroid cancer group—papillary and follicular thyroid carcinomas—from the less frequent but clinically aggressive histologic types, such as poorly differentiated and anaplastic thyroid carcinomas. Anaplastic thyroid carcinoma is an uncommon carcinoma representing 1 to 4% of all thyroid cancers. This carcinoma is most common in females of the eight decades [1]. Causative factors for anaplastic carcinoma remain unknown, although they might—at least partly—overlap with those of well-differentiated thyroid carcinomas, as suggested by the frequent co-occurrence of well-differentiated components in anaplastic carcinoma cases. Poorly differentiated thyroid carcinoma is even rarer than anaplastic thyroid carcinoma and its incidence varies worldwide, as a possible consequence of environmental factors and classification criteria [2]. In a large series from a single institution, the incidence of poorly differentiated thyroid carcinoma is 3% of primary thyroid carcinoma and slightly more half the incidence of anaplastic thyroid carcinoma [3]. This carcinoma is more common in females of the sixth decades. A history of longstanding goiter (> 10 years) was noted in 27% of patients with poorly differentiated carcinoma and 24% of patients with anaplastic carcinoma [3].

In the latest WHO classification [4], the main definitional parameters for classification of these aggressive variants have not been substantially modified. Although novel diagnostic terms (such as non-invasive follicular tumor with papillary-type nuclei—NIFTP) have been introduced and some redefined (such as oncocytic tumors are now regarded as a separate group of tumors), the criteria for tumor classification have remained the same in spite of remarkable advances in the knowledge of the molecular landscape of thyroid cancer.

The better understanding of this landscape largely stems from the application of high throughput technologies. From the molecular standpoint, tumor groups are associated with differentiation, invasive properties, and particularly with cancer architecture—papillary versus follicular. The recognition of *RAS*-like and *BRAF* V600E-like molecular profiles—formalized by the work by the The Cancer Genome Atlas (TCGA) [5]—has linked the former to follicular and the latter to papillary growth. For example, *RAS* gene mutations are strongly associated with follicular architecture, irrespective of the presence or absence of invasion and of the nuclear features of neoplastic cells: *RAS* mutation prevalence is similar in follicular adenoma, follicular carcinoma, and follicular variant papillary carcinoma. The same holds true for other less frequent molecular alterations such as *BRAF* non-V600E mutations (e.g., *BRAF* K601E) or *PTEN* mutations, all recognized in tumors with follicular architecture at similar prevalence rates among the different histologic types. On the other end, *BRAF* V600E and similar molecular alterations such as *RET* and *NTRK* rearrangement are the defining molecular markers for “conventional” papillary thyroid carcinoma, i.e., those characterized by the presence of neoplastic papillae. In poorly differentiated and anaplastic thyroid carcinoma, *RAS*-like and *BRAF* V600E-like signatures are partially retained since a subset of cases is characterized by mutations of *BRAF* and *RAS* genes at prevalence rates not very different from those of well-differentiated papillary and follicular carcinoma. By contrast, *TERT* promoter mutations are exclusively associated with malignancy, are invariably associated with invasive growth in papillary and follicular carcinomas, and their prevalence increases in poorly differentiated and anaplastic thyroid carcinomas.

**Fig. 1:**
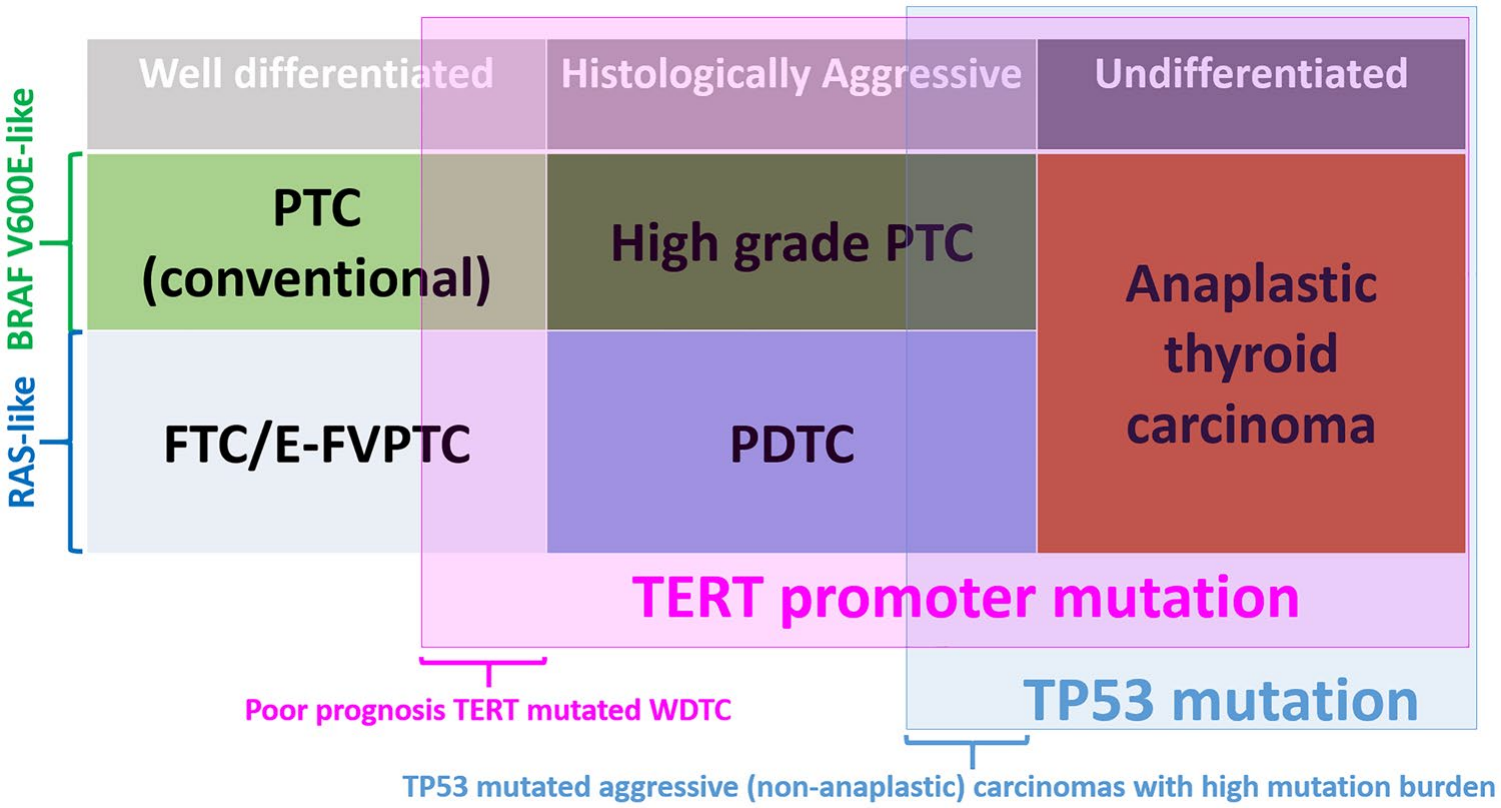
Schematic representation of the overlap between tumor type, *TERT* promoter, and *TP53* mutation. *TERT* promoter mutation is a powerful marker of poor prognosis irrespective of tumor histotype. *TP53* mutation is associated with high mutation burden but the correlation with poor prognosis is not independent of the tumor histotype. PTC: papillary thyroid carcinoma; FTC: follicular thyroid carcinoma; E-FVPTC: encapsulated follicular variant papillary thyroid carcinoma; PDTC: poorly differentiated thyroid carcinoma; WDTC: well-differentiated thyroid carcinoma. *BRAF* V600E-like and *RAS*-like: molecular profile according to the Cancer Genome Atlas (TCGA), Integrated Genomic Characterization of Papillary Thyroid Carcinoma [5]. Pink shading indicates those tumor types with a high proportion of *TERT* promoter mutations. Blue shading indicates those tumor types with a high proportion of *TP53* mutations

It is important to recognize that the correlation of molecular alterations with tumor pathology is affected by classification criteria. Anaplastic carcinoma is considered the end point of follicular cell-derived cancer progression and—despite its heterogeneous morphology—is defined by the presence of high-grade features and lack of follicular cell differentiation [1]. The criteria for poorly differentiated carcinoma are still somewhat controversial [6–8]. Its features—as outlined in the Turin consensus [9]—define the prototype of a thyroid carcinoma that is both high grade and poorly differentiated [6] and have been embraced by the WHO classification [4]. Turin criteria should be used in an algorithmic approach and include (a) presence of a solid/trabecular/insular pattern of growth in a malignant/invasive tumor, (b) absence of the conventional nuclear features of papillary carcinoma, and (c) presence of at least one of the following features: convoluted nuclei, mitotic activity ≥ 3 × 10 HPF; tumor necrosis [9]. Proliferative grading—as used by the group of Memorial Sloan Kettering Cancer Center in the USA to designate tumors “poorly differentiated” [10]—also includes aggressive, potentially lethal forms of thyroid carcinoma that still retain histologic differentiation (Fig. 1). The vast majority of them are high-grade papillary carcinomas, which are thyroid carcinomas maintaining the typical nuclear features of papillary carcinoma together with increased mitotic activity (> 5 mitoses per 10 HPF) and/or necrosis [11]. It is also important to note that even minor components of either poorly differentiated or anaplastic thyroid carcinomas in an otherwise well-differentiated carcinoma are impacting negatively on patients’ prognosis, and these should be mentioned in the pathological report. In fact, the prognosis of patients with thyroid carcinomas having a component of either poorly differentiated or anaplastic thyroid carcinoma at the threshold of 10% were shown to bear a prognosis similar to predominant poorly differentiated/anaplastic ones [12, 13].

Finally, data on the molecular background of poorly differentiated and anaplastic thyroid cancers are informative not only to depict histologic type-specific characteristics, but also to unravel the mechanisms of thyroid tumor progression and to identify potential therapeutic targets in a group of neoplasms whose response to radio-iodine therapy is low (in poorly differentiated carcinoma) or absent (in anaplastic carcinoma). The impact of molecular pathology in these tumors is therefore multifaceted and bears diagnostic, prognostic, and predictive implications although its use in the clinical practice is neither definitely established nor precisely coded in current guidelines.

## Genomic Profiling

In the recent past, there has been a flurry of studies that have analyzed in depth the genomic profiling of aggressive forms of thyroid carcinoma, including poorly differentiated and anaplastic thyroid carcinomas. The common driver mutations and somatic genetic alterations of poorly differentiated and anaplastic thyroid carcinomas compared with those of well-differentiated histologic types are summarized in Table 1. In the table, those pertinent to poorly differentiated carcinoma are defined according to the Turin criteria, and those of papillary carcinoma may include some of the uncommon papillary carcinomas of high proliferative grade.

**Table 1:** Common driver mutations and somatic genetic alterations in poorly differentiated and anaplastic thyroid carcinomas compared with differentiated thyroid carcinoma^*a*^

Differentiated thyroid carcinoma		Poorly differentiated thyroid carcinoma^*b*^	Anaplastic thyroid carcinoma
Follicular carcinoma^*c*^	Papillary carcinoma (conventional types)		
*RAS* (30–50%)^*d*^ *PAX8/PPPARG* (10–30%) *TERT* (10–35%)^*f*^ *PIK3CA* (0–10%) *PTEN* (0–10%) Can be genetically unstable and aneuploid, median number of mutations: up to 5^*i*^ *RAS*-like TCGA molecular profile^j^	*BRAF* V600E (40–80%) *RET/PTC* (5–20%)^*e*^ *TERT* (5–15%)^*f*^ *RAS* (0–10%)^*d*^ *NTRK* rearrangement (0–10%)^g^ Genetically stable, median number of mutations: 1 ± 1^*i*^ *BRAF* V600E-like TCGA molecular profile^j^	*RAS* (20–50%)^*d*^ *TERT* (20–50%)^*f*^ *TP53* (10–35%) *BRAF* V600E (1–10%)^*i*^ *PTEN* (5–20%) *PIK3CA* (0–15%) *EIF1AX* (5–15%)^*h*^ *ALK* rearrangement (0–10) Genetically unstable, aneuploid, median number of mutations: 2 ± 3^*i*^ Typically have *RAS*-like TCGA molecular profile^j^	*TP53* (40–80%) *TERT* (30–75%)^*f*^ *RAS* (10–50%)^*d*^ *BRAF* V600E (10–50%) *PIK3CA* (5–25%) *PTEN* (10–15%) *EIF1AX* (5–15%)^*h*^ *ALK* rearrangement (0–10) Genetically unstable, highly aneuploid, median number of mutations: 6 ± 5^*i*^ May have *RAS*-like *or BRAF* V600E-like TCGA molecular profile^j^

^*a*^Main molecular alterations, in parentheses the estimated mutation prevalence range

^*b*^Poorly differentiated thyroid carcinoma according to Turin criteria [9]

^*c*^Encapsulated follicular variant papillary thyroid carcinoma, with invasion or without invasion (NIFTP) have molecular alterations like that of follicular thyroid carcinoma

^*d*^Mutations in *N-, H, K-RAS*; *N-RAS* is the gene most frequently mutated

^*e*^The prevalence of *RET/PTC* is higher in children and much higher in radiation-associated papillary thyroid carcinomas

^*f*^*TERT* promoter mutations: C228T [c.-124G > A] and C250T [c.-146G > A]

^*g*^The prevalence of NTRK rearrangements is variably reported between 0 and 5% for NTRK1 and NTRK3 in most series from non-radiation associated papillary carcinoma in adult patients; the prevalence is higher in children and young patients, and in radiation-associated papillary carcinoma

^*h*^EIF1AX mutations occur in RAS mutated tumors

^*i*^The mutation burden per tumor is estimated from: Cancer Genome Atlas Research Network [5] Landa I et al. [15] Pozdeyev N et al. [20]

^*j*^The Cancer Genome Atlas (*TCGA*), Integrated Genomic Characterization of Papillary Thyroid Carcinoma [5]

Most of the data from the genomic profile of aggressive forms of thyroid cancer—poorly differentiated carcinoma, anaplastic carcinoma, high-grade papillary carcinoma, and advanced forms of thyroid cancer in general—are quite consistent [14–29].

Data converge on several important points:

- Genetic alterations include “Early” and “Late” molecular events.
- “Early” changes are found in combination with “Late” alterations, consistent with a general model of multi-step progression from well differentiated to poorly differentiated to anaplastic thyroid carcinoma; in cases where poorly differentiated or anaplastic areas are associated with a well differentiated component, “Early” alterations are identified in both areas, while “Late” changes are restricted to the less differentiated portions of the tumor [29–31].
- “Early” driver events are mostly *RAS* and *BRAF* mutations, thus aggressive thyroid cancers retain *BRAF* V600E-like or *RAS*-like signatures. *RAS*-activating mutations have a high prevalence in poorly differentiated thyroid carcinoma, indicating that most develop from follicular carcinoma or from follicular variants of papillary carcinoma.
- The most frequent “Late” changes associated with dedifferentiation and progression are somatic mutations of *TP53*, *TERT* promoter and dysregulation of the PI3K/PTEN/AKT pathway. Mutations of *CDKN2A*, SWI/SNF (switch/sucrose non-fermentable) chromatin remodeling complex genes (*ARID1A*, *ARID1B*, *ARID2*, *ARID5B*, *SMARCB1*, *PBRM1*, *ATRX*), histone methyltransferase genes (*KMT2A*, *KMT2C*, *KMT2D*, *SETD2*), and DNA mismatch repair (MMR) genes (*MSH2*, *MSH6*, and *MLH1*) are associated more frequently with anaplastic thyroid carcinoma as opposed to other aggressive forms of thyroid cancer [19, 28].
- The number of mutations per tumor increases from well differentiated to poorly differentiated to anaplastic thyroid carcinoma. Mutation burden is highest in anaplastic thyroid carcinoma, lowest in papillary thyroid carcinoma, and intermediate in aggressive/advanced papillary and follicular thyroid carcinomas.
- *TERT* promoter mutations are more frequent and have higher mutated allelic fraction in poorly differentiated, anaplastic, and in aggressive/advanced thyroid carcinomas (including high-grade variants of papillary carcinoma) compared with well-differentiated thyroid carcinomas.
- *TERT* promoter mutations are a powerful marker of poor prognosis, independent of tumor histological type. The prognosis is most unfavorable when *TERT* promoter is co-mutated with *BRAF* V600E or *RAS* [19, 28]. Aggressive/advanced papillary carcinomas, many of which are histologically high grade, have at last one of three genetic alterations: duplication of chromosome 1q, duplication of chromosome 5p harboring the *TERT* genomic locus and *TERT* promoter mutation (THYT1 signature) [18].
- *TP53* mutation has the highest prevalence in anaplastic thyroid carcinoma compared with all forms of advanced/aggressive thyroid carcinoma, including both poorly differentiated and high-grade papillary thyroid carcinoma. Unlike *TERT* promoter mutation, the impact *TP53* mutation on survival is not independent of tumor histological type. In combined tumors, it segregates with the anaplastic component [19, 21, 29]
- Rearrangements—such as *RET/PTC*, *NTRK1* and 3, *PAX8-PPRG*—are not rare in well-differentiated thyroid carcinomas but are uncommon in poorly differentiated and anaplastic thyroid carcinomas [19, 28].

The role of *TERT* promoter mutations as “Late” events is still a matter of exploration. Their prevalence is invariably low in well differentiated neoplasms, with special reference to follicular patterned lesions with no or minimal vascular invasion. In a study, *TERT* promoter mutations were absent in 60 follicular adenomas and in 16 NIFTP cases, but present in 3 of 83 papillary carcinomas, follicular variant, and in 3 of 29 cases of minimally invasive follicular carcinomas [32]. However, *TERT* promoter mutations have been described as subclonal events in a metastatic follicular carcinoma with a wide spatial heterogeneity [33]. Such intratumoral heterogeneity might influence detection rates and prevalence data. Moreover, it is impacting on the hypotheses of molecular progression, suggesting that *TERT* promoter mutations can anticipate clinical or morphological signs of progression. A propensity of thyroid cancer cells to accumulate *TERT* promoter mutations, as additional molecular events other than early genomic drivers, is also supported by in vitro data reporting a very high frequency in papillary and follicular thyroid carcinoma-derived cell lines [34].

A special comment is deserved to two specific topics whose clinical impact also includes potential therapeutic implications. First, there has been a growing interest in the analysis of gene fusions in aggressive thyroid cancer types since—although rare in terms of prevalence—they represent a potential therapeutic target. In the most extensive molecular studies performed so far, gene fusions have been detected in 11 of 52 cases of poorly differentiated thyroid carcinoma (*RET* rearrangement in 5 cases, *ALK* rearrangement in 3 cases, *PAX8/PPARG* gene fusion in 3 cases) [15] and in 5 of 107 cases of anaplastic thyroid carcinoma (*RET* rearrangement in 2 cases, *NTRK* rearrangement in 3 cases) [28]. Moreover, a high prevalence (16%) of poorly differentiated thyroid carcinomas has been observed in a series of *ALK*-fused thyroid cancers [35]. Even more interestingly from a pathogenetic point of view, the presence of the *STRN-ALK* gene fusion has been shown to promote progression and loss of differentiation in a mouse model of thyroid cancer [36].

The second issue is related to the presence of MMR defects and microsatellite instability. As mentioned above, poorly differentiated and anaplastic thyroid cancers feature progressively increased mutational burden and mutations in MMR genes in whole genome sequencing studies. In addition, subclonal analysis of a thyroid cancer with follicular, poorly differentiated, and anaplastic components detected microsatellite instability in the two latter components despite *MSH2* mutation was already present in the follicular carcinoma component [37]. However, data on large series as well as correlations of MMR status with clinical or pathological characteristics are lacking. Moreover, the few studies available report discordant results, as also outlined in a recent report [38]. Thus, the real prevalence of the MMR status in poorly differentiated and anaplastic thyroid carcinomas still needs to be clearly outlined. Indeed, although the estimated prevalence is low, the MMR status has a major impact in defining potential therapeutic implications in terms of immunotherapy strategies and to promote a patient-tailored design of therapy. Moreover, recent gene expression studies are showing that poorly differentiated and anaplastic thyroid carcinomas possess specific tumor-immune profiles, with special reference to activation of M2-class macrophages and PD-L1 protein expression [20, 39, 40]. Immune-related tumor expression profiles are apparently associated with individual tumor properties within each histologic type (i.e., the presence of oncocytic features in poorly differentiated carcinoma), which should be characterized more extensively since typing a subset of cases may potentially benefit patients with the use of immunotherapy.

Finally, the molecular landscape of aggressive thyroid cancer histological types in pediatric patients may be different from that of the adult population. Anaplastic carcinoma is extremely rare in children. In a recent study, Chernock et al. [41] identified somatic *DICER1* mutations in five of six poorly differentiated carcinomas of children and adolescents diagnosed according to Turin criteria, in the absence of the mutations commonly found in thyroid tumors (*BRAF*, *RAS*, *TERT*, *RET/PTC* etc.). This indicates that these tumors may be genetically distinct from histologically similar adult-onset carcinomas. Importantly, a germline pathogenic *DICER1* mutation was identified in one of the five *DICER1*-mutated cases. Thus, poorly differentiated thyroid carcinoma should be included in the spectrum of thyroid lesions associated with *DICER1* syndrome and genetic counselling may be considered for all young patients diagnosed with poorly differentiated thyroid carcinoma. However, *DICER1* mutations are also associated with well-differentiated carcinomas in the pediatric population [42]. Therefore, *DICER1* mutations may represent “Early” events in this specific population whereas progression to poorly differentiated carcinoma may be associated to additional “Late” events, in a model similar to the adult counterpart. In this respect, in the Chernock et al. series, *DICER1*-co-mutated genes included *TP53*, *ATM*, and *ARID1A* (among others) [41], all known to have an increased prevalence in poorly differentiated carcinomas in adults.

## Epigenetic Modifications

**Fig. 2:**
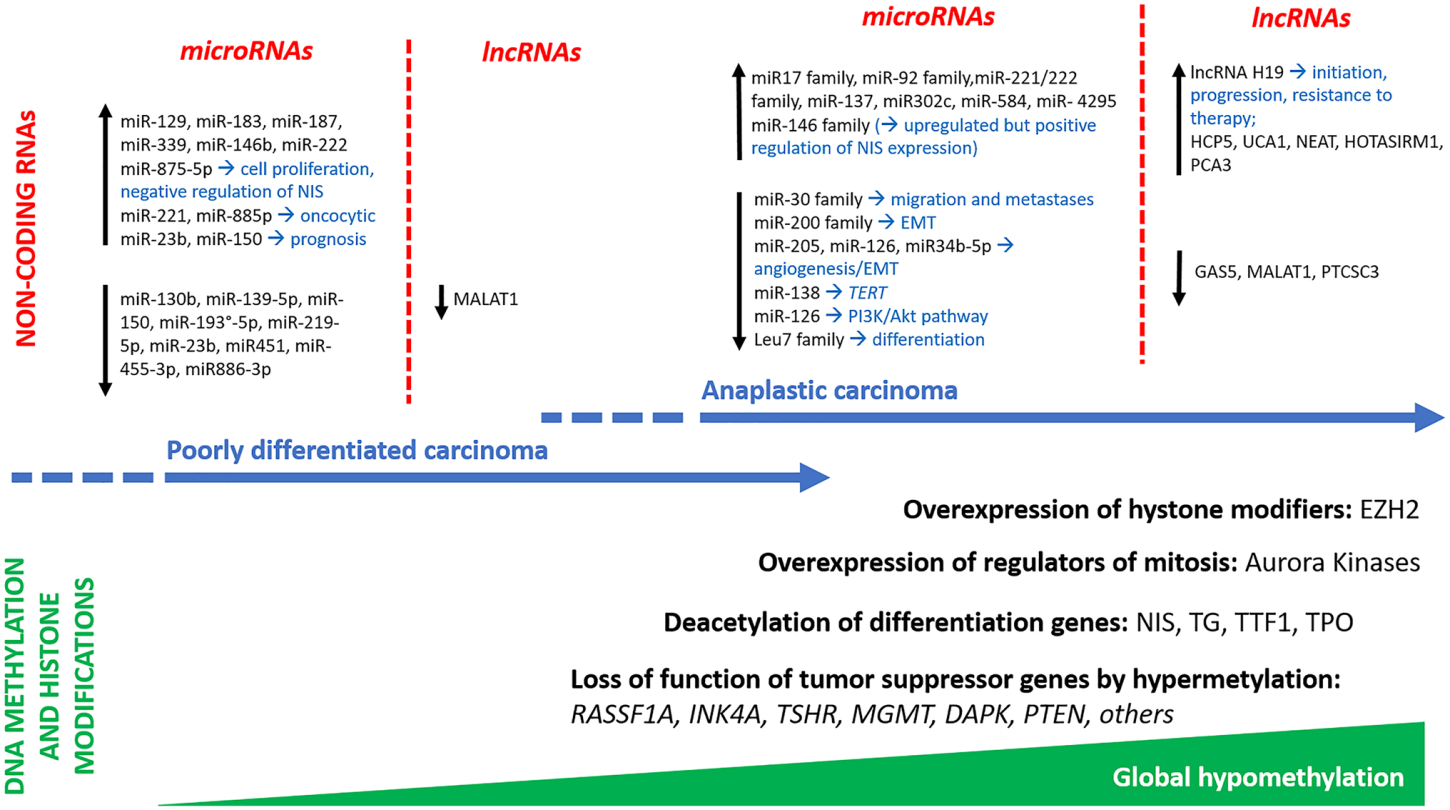
Summary of epigenetic hallmarks of poorly differentiated and anaplastic carcinoma

The genomic map of poorly differentiated and anaplastic thyroid carcinomas is not fully explaining the pathogenesis of these tumor types, their biological and clinical properties, or their pathways of progression. Although the prevalence of specific gene alterations increases proportionally to the loss of differentiation and aggressiveness, most gene alterations are shared by well differentiated and poorly differentiated/anaplastic carcinomas; thus, they probably represent initiating events in the oncogenic process. In this context, epigenetic regulatory mechanisms possibly represent major complementary players in the progression to both poorly differentiated and anaplastic thyroid carcinoma (Fig. 2). The impact of epigenetic mechanisms is also claimed by the few data on gene expression signatures available. In fact, well-differentiated thyroid carcinomas mostly display gene expression features consistent with two major signatures, *BRAF-* and *RAS*-like. However, in aggressive carcinomas—in particular anaplastic carcinoma—transcriptomic profiles seem to be distinctive of the tumor types per se, irrespective of the genotype [24]. Moreover, genomic data and transcriptomic profiling show that epigenetic mechanisms are involved in aggressive types of thyroid cancer. In fact, histone methyltransferase genes (*KMT2A*, *KMT2C*, *KMT2D*, and *SETD2*) are increasingly impaired in poorly differentiated and anaplastic thyroid carcinomas [15]. Nevertheless, the increased expression of histone methyltransferase genes that is associated with the pattern of dedifferentiation observed in poorly differentiated and anaplastic thyroid carcinomas needs to be further investigated. Similar trends hold true for genes belonging to the SWI/SNF complex, a chromatin remodeling complex which is active in nucleosome-remodeling.

### DNA Methylation and Histone Modifications

In cancer, two main alterations in DNA methylation are distinguished. On the one hand, it is common to find locus-specific hypermethylation that mainly affects regulatory elements such as promoters, which can lead to silencing of tumor suppressor genes or genes that are important for cellular function such as DNA repair and apoptosis. On the other hand, tumor cells often show a global DNA hypomethylation, which affects extensive domains of the genome and promotes genomic instability. Most available studies on gene methylation status in thyroid cancer are based on the analysis of well-differentiated thyroid carcinomas, mostly papillary thyroid carcinomas. They show consistent data on the association of specific methylation signatures with adverse prognostic parameters [43], without identifying valid biomarkers. A few studies have analyzed the methylation status of specific genes in poorly differentiated and anaplastic thyroid carcinomas. Their informative impact is limited but the prevalence of alterations is generally higher when compared with that of differentiated thyroid carcinoma. For example, in one study, *RASSF1* silencing by methylation was detected in the only poorly differentiated carcinoma analyzed and in 78% of anaplastic carcinomas, which had the highest rate among all other histologic types tested [44]. Other tumor suppressor genes, such as p16(*INK4A*), *TSHR*, *MGMT*, *DAPK*, *ESR1*, *ESR2*, *RARbeta*, *PTEN*, *CD26*, *SLC5A8*, and *UCHL1*, are frequently methylated in anaplastic thyroid carcinomas as compared with well-differentiated carcinomas [45]. Interestingly, epigenetic changes have also been associated with specific genomic alterations. In particular, the presence of demethylation (expressed as the decrease of 5-hydroxymethylcytosine consequent to the downregulation of ten-eleven translocation family of 5-mC hydroxylases) has been associated with the presence of *TERT* promoter mutations and anaplastic thyroid cancer histology [46].

Histone methylation modifiers, such as enhancer of zeste homolog 2 (EZH2), have been shown to be overexpressed in anaplastic thyroid carcinoma cells, suppressing PAX-8 transcription [47]. In addition, Aurora group members A, B, and C, which act as regulators of mitotic events by controlling the histone H3 phosphorylation and chromatin remodeling process, are overexpressed in anaplastic thyroid carcinoma [48]. Concerning global hypomethylation, poorly differentiated and anaplastic thyroid carcinomas (16 cases in total) have been shown to present a hypomethylated pattern in 93.8% of cases, as compared with 3.6% of low-risk well-differentiated carcinomas and 42.4% of metastatic well-differentiated carcinomas, irrespective of the genotype [49]. Histone deacetylation can also epigenetically alter gene expression. Among genes that are regulated by deacetylation in thyroid cancer are “differentiation genes”, such as sodium-iodide symporter (NIS), thyroglobulin, TTF-1, and thyroid peroxidase [50]. However, despite preclinical evidence of a potential role for inhibitors of deacetylation in promoting re-differentiation of anaplastic thyroid carcinoma [51], there neither currently is an established clinical impact for this potential therapeutic strategy nor are there data on poorly differentiated thyroid carcinoma.

### Non-coding RNAs

Non-protein-producing RNAs, including microRNAs (miRNAs) and long non-coding RNAs (lncRNAs), are potential targets to treat poorly differentiated and anaplastic thyroid carcinoma and likely play an important role in their pathogenesis [52]. In addition, small interfering RNAs (siRNA) mediate transcriptional gene silencing in cells through DNA methylation and histone modification.

## MicroRNAs

It is well established that improper epigenetic regulation by microRNAs contributes to tumor progression in poorly differentiated and anaplastic thyroid carcinomas [53]. Up- or downregulation of microRNAs can influence the tumorigenic outcome depending on the role(s) of the target genes on vital signaling processes. While aberrant expression of hundreds of microRNAs has been identified in several types of thyroid carcinoma, very few have been found to be exclusively dysregulated in either poorly differentiated or anaplastic carcinomas, and a small subset of them have been found to be de-regulated in both histologic types [54]. The biological functions of these microRNAs are heterogeneous and interconnected, as are their potential gene targets and pathways. MicroRNAs are generally downregulated rather than upregulated in poorly differentiated and anaplastic carcinomas [55].

In anaplastic thyroid carcinoma, several microRNAs are known to be dysregulated [52]. For instance, the miR-30 family of tumor suppressor microRNAs has been found to be downregulated [56], and miR-30a overexpression has been shown to suppress migration, tumor spreading, and metastasis in vitro and in vivo [57]. Downregulation of some microRNAs may increase the expression of oncogenes, such as miR-138 that targets *TERT* and is downregulated in anaplastic carcinoma [58]. Epithelial to mesenchymal transition (EMT) may also be affected by microRNAs. MiR-200 family members, reported to be under-expressed in anaplastic thyroid cancer, possibly regulate EMT through the modulation of TGFβ receptor 1 and Rho/ROCK-mediated signaling [59, 60]. miR-205 targets angiogenesis and EMT concurrently in anaplastic thyroid carcinoma [61]. Moreover, differentiation of thyroid cancer cells—as promoted by the expression of transcription factors—might be also influenced by microRNAs. For example, Let-7 microRNA family members are positive regulators of thyroid transcription factor-1. Multiple studies showed a marked decrease in the expression of let-7a, let-7c, let-7d, let-7f, let-7g, let-7i, miR-126, miR-205, and miR-34b-5p in anaplastic thyroid carcinoma [56, 59]. The reduced expression of these miRNA is consistent with a tumor suppressor function and they may have a role in the pathogenesis of anaplastic carcinoma. For instance, introduction of exogenous miR-126, miR-205, or miR-34b-5p into anaplastic thyroid carcinoma cells results in a significant reduction of VEGF-A protein expression indicating that these miRNAs may negatively regulate vascular proliferation in anaplastic carcinoma [62–64]. In addition, miR-126 has been reported as proliferation suppressor targeting the PIK3R2 gene and repressing P13K-AKT proliferation signaling pathway [65]. In the poorly differentiated carcinoma subgroup, miR-130b, -139-5p, -150, -193a-5p, -219-5p, -23b, -451, -455-3p, and miR-886-3p have been shown to be downregulated as compared with normal tissue [66].

Concerning microRNA upregulation, anaplastic carcinoma is characterized by increased expression of miR-17-92 cluster, miR-137, miR-146 family, miR-221/222 families, miR-302c, miR-584, and miR-4295 [67, 68]. The more frequently upregulated microRNAs in poorly differentiated carcinoma versus normal tissue are miR-129, miR-183, miR-187, miR-339, miR-146b, miR-221, and miR-222, the last three in common with the anaplastic carcinoma group [66, 67]. However, miR-221 and miR-222 have been found to be downregulated as compared with well-differentiated thyroid carcinomas [66]; thus, their role in thyroid cancer progression is still to be clarified. In addition, some microRNAs have also been associated to peculiar clinical and pathological features in poorly differentiated thyroid carcinomas. In fact, upregulation of miR-221 and miR-885-5p has been reported in oncocytic variant as compared with conventional poorly differentiated carcinomas, whereas upregulation of miR-23b and miR-150 has been correlated with tumor relapse and tumor-specific death, respectively [66].

Finally, the recognition that selected microRNAs can modulate the expression of NIS in dedifferentiated thyroid cancer cells is of special interest. For example, miR-146b contributes to the recovery of radioiodine sensitivity in dedifferentiated cells by positively regulating NIS in vitro [69]. Moreover, upregulation of miR-875-5p has been shown to induce cell proliferation and to reduce apoptosis and radioiodine uptake through down-regulation of NIS in the poorly differentiated thyroid carcinoma cells (cell line WRO) in vitro and in an orthotopic model [70]. Apart from diagnostic or pathogenetic issues, these preliminary data, as also discussed above for histone deacetylation modifiers, are opening avenues to specific therapeutic interventions aimed at restoring radioiodine responsiveness in dedifferentiated thyroid cancer cells.

## Long Non-coding RNA

Long non-coding RNAs (lncRNAs) are non-protein-coding transcripts more than 200 nucleotides in length. LncRNAs regulate target gene expression at the transcriptional level by recruiting DNA methyltransferases to modify chromatin conformation or by complementary sequence-specific mechanisms that affect the mRNA splicing at the post-transcriptional level. The dysregulation of lncRNA in thyroid cancer has been extensively studied in different histologic types, including anaplastic thyroid carcinoma, whereas no specific data are available for poorly differentiated thyroid carcinoma. Thus, several lncRNA have been shown to be up or downregulated in anaplastic cancer, possessing either tumor suppressive or oncogenic activity. Extensive reviews of lncRNAs regulation in anaplastic carcinomas are available in the recent literature [52, 71]. High expression of lncRNA *H19 *gene has been shown to promote initiation, progression, and therapy resistance of anaplastic carcinoma [72]. Moreover, in this same study, targeting H19 inhibited tumor metastases by approximately eight-fold in comparison with that of controls, suggesting that H19 may be a potential target for molecular therapy in anaplastic cancer patients. However, the target gene(s) of H19 are still unknown. Several other lncRNAs are active as oncogenes (upregulated) or tumor suppressors (downregulated) in anaplastic cancer. Restricting the list to those that have been analyzed in tumor tissue samples HCP5 [73], UCA1 [74], NEAT1 [75], HOTAIRM1, and PCA3 [76] are oncogenic, whereas GAS5 [77], MALAT1 (in this case, poorly differentiated carcinoma samples have also been analyzed) [78] and PTCSC3 [79] are tumor suppressors. Their targets are either microRNAs or coding genes that contribute to the modulation of several functions, including cell cycle and growth, invasion, migration, apoptosis, differentiation, autophagy, responsiveness to chemotherapy, and cell stemness. However, despite the relatively large availability of data and the wide impact in regulating tumor-specific cellular functions, the role of lncRNAs as clinical biomarkers has still to be defined.

## Molecular Mechanisms of Progression from Well-Differentiated to Poorly and Anaplastic Thyroid Carcinomas

**Fig. 3:**
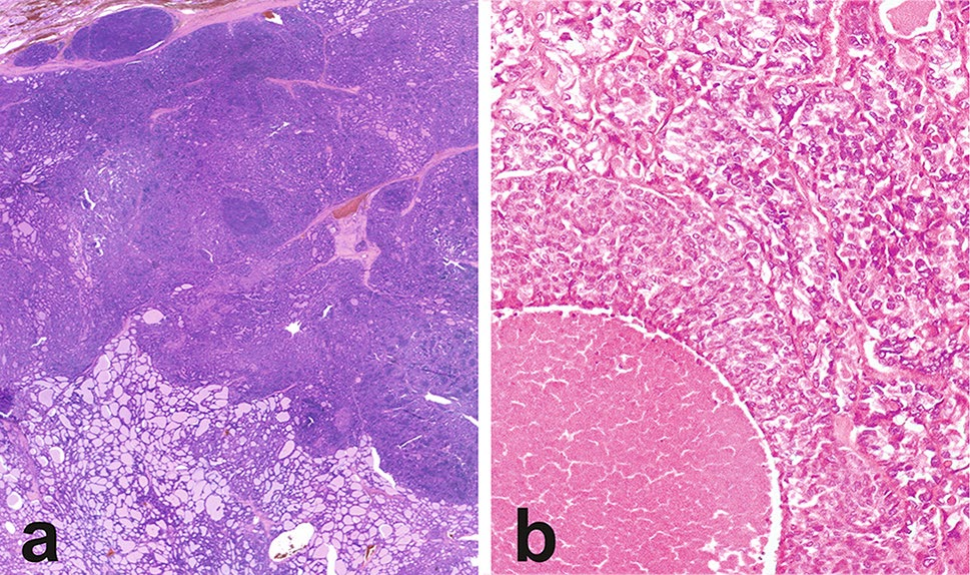
Patterns of coexistence of poorly differentiated and wel-differentiated thyroid carcinoma components. Poorly differentiated carcinoma areas with solid growth and vascular invasion at the periphery of a follicular carcinoma (**a**). Poorly differentiated thyroid carcinoma with solid growth and comedo necrosis associated with a papillary carcinoma with microfollicular architecture (**b**)

Poorly differentiated and anaplastic thyroid carcinomas may arise either de novo or from/in combination with well-differentiated thyroid carcinomas (Fig. 3). In a large series by one of us, co-existing well-differentiated thyroid carcinoma were noted in 59% of patients with poorly differentiated thyroid carcinoma and 39% of anaplastic thyroid carcinoma [3]. The molecular background responsible for these two situations is not fully elucidated. In fact, the genomic landscape of cases supposed to arise de novo as compared with those undergoing de-differentiation has not been explored in detail, especially because of the difficulty to clearly define the former group. Old ploidy studies on co-existent anaplastic and well-differentiated thyroid carcinomas claimed that aneuploid DNA pattern was almost exclusive of anaplastic thyroid carcinoma. Therefore, the authors suggested that the majority of anaplastic thyroid carcinomas arise de novo rather than through clonal transformation of well-differentiated thyroid carcinomas [80]. However, the genetic alterations in cases of poorly differentiated thyroid carcinomas arising within a nodule of goiter, thus paradigmatic of de novo origin, are unspecific (i.e., *NRAS* mutations) [81].

**Fig. 4:**
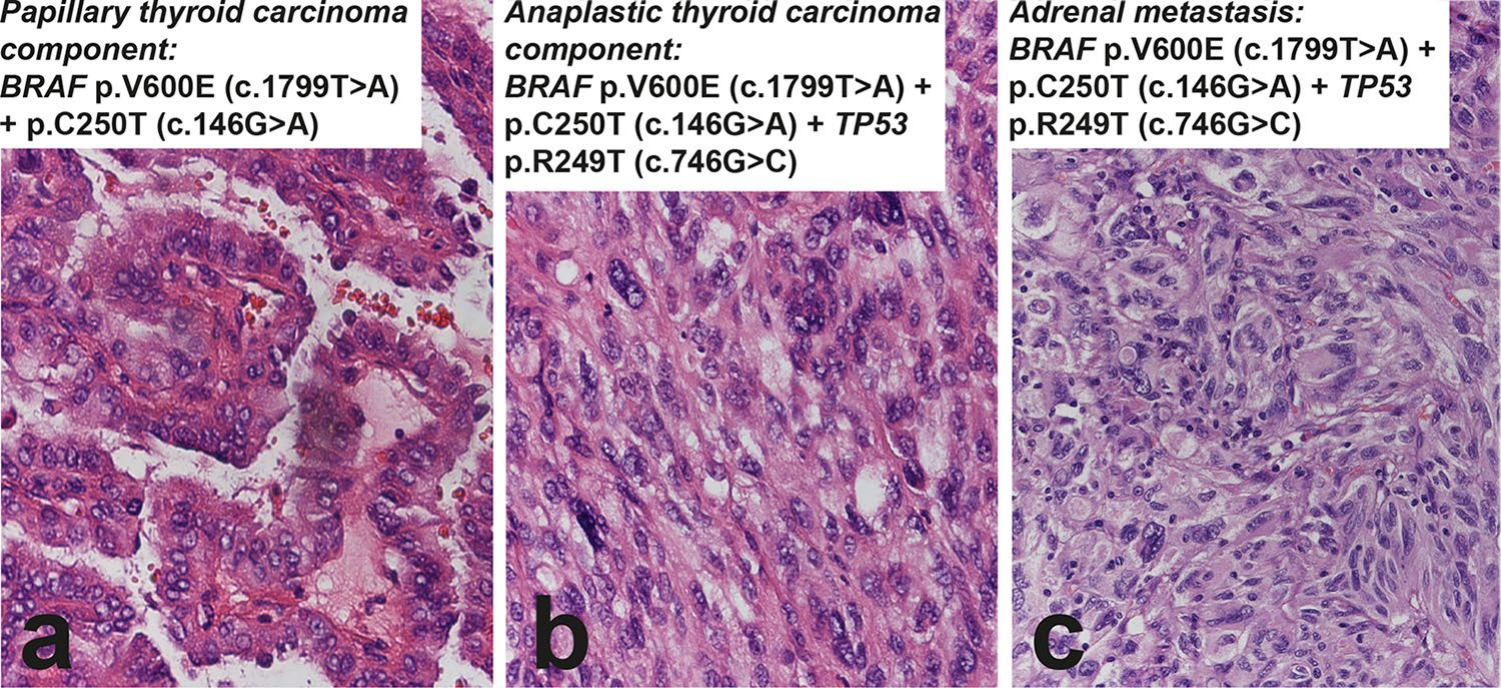
Sixty-eight-year-old woman with thyroid carcinoma and adrenal metastasis. The thyroid carcinoma has a conventional papillary carcinoma component that is BRAF p.V600E and TERT promoter mutated (C250T) (**a**), associated to a small focus of anaplastic thyroid carcinoma that in addition to the same BRAF and TERT mutations carries TP53 R249T (**b**). The adrenal metastasis (**c**) demonstrates morphology and molecular features (BRAF, TERT promoter, and TP53 mutations) identical to the anaplastic thyroid carcinoma component in the primary tumor

On the other hand, the precise mechanisms of clonal evolution, tumor progression, and dedifferentiation in cases with co-existent well-differentiated and poorly differentiated/anaplastic carcinoma components are poorly understood. In anaplastic thyroid carcinoma, the quest for possible well-differentiated precursors has been more extensive and four types of anaplastic cancers have been proposed after extensive genomic analysis [20]. Type 1 is composed of *BRAF* V600E mutated tumors likely evolved from papillary thyroid carcinoma. Type 2 is composed of tumors with *NRAS* mutation likely evolved from follicular patterned tumors. Type 3 is a cluster composed of high mutation burden tumors with MMR signature and mutations in *MSH2* and *MLH1* genes. In these tumors, *PTEN* mutation frequently coexists with NF1 and RB1 mutations. Amplification of chromosome 4q12 (including *KIT*, *KDR*, *PDGFR* genes) or 9p24.1 (including the immune evasion genes *CD274*, *PDCD1LG2*, *JAK2*) are common. The frequent occurrence of *RAS* mutations suggests that these tumors may have evolved from a subset of follicular patterned carcinomas. Type 4 is mixed without a defined well-differentiated precursor. Genes associated with aggressive phenotype, such as *TERT*, are generally mutated in both well-differentiated and anaplastic components [29], suggesting that *TERT*-mutated carcinomas that are histologically well differentiated possess an intrinsic predisposition to dedifferentiation. By contrast, as already mentioned above, *TP53* mutations occur almost exclusively in the anaplastic component (Fig. 4).

Data on the molecular heterogeneity of poorly differentiated carcinomas associated with well-differentiated components are lacking. In a recent study, the analysis of the molecular landscape of 41 poorly differentiated thyroid carcinomas revealed distinct genomic events in cases with papillary or follicular associated components, with higher prevalence of *BRAF* and *PIK3CA* mutations as well as *RET* and *NTRK* fusions in the former, and higher prevalence of *TERT* mutations with no fusions in the latter [27]. However, subclonal molecular analysis of well and poorly differentiated thyroid carcinoma components was not performed in these cases.

## Current and Potential Impact of Molecular Biomarkers

**Fig. 5:**
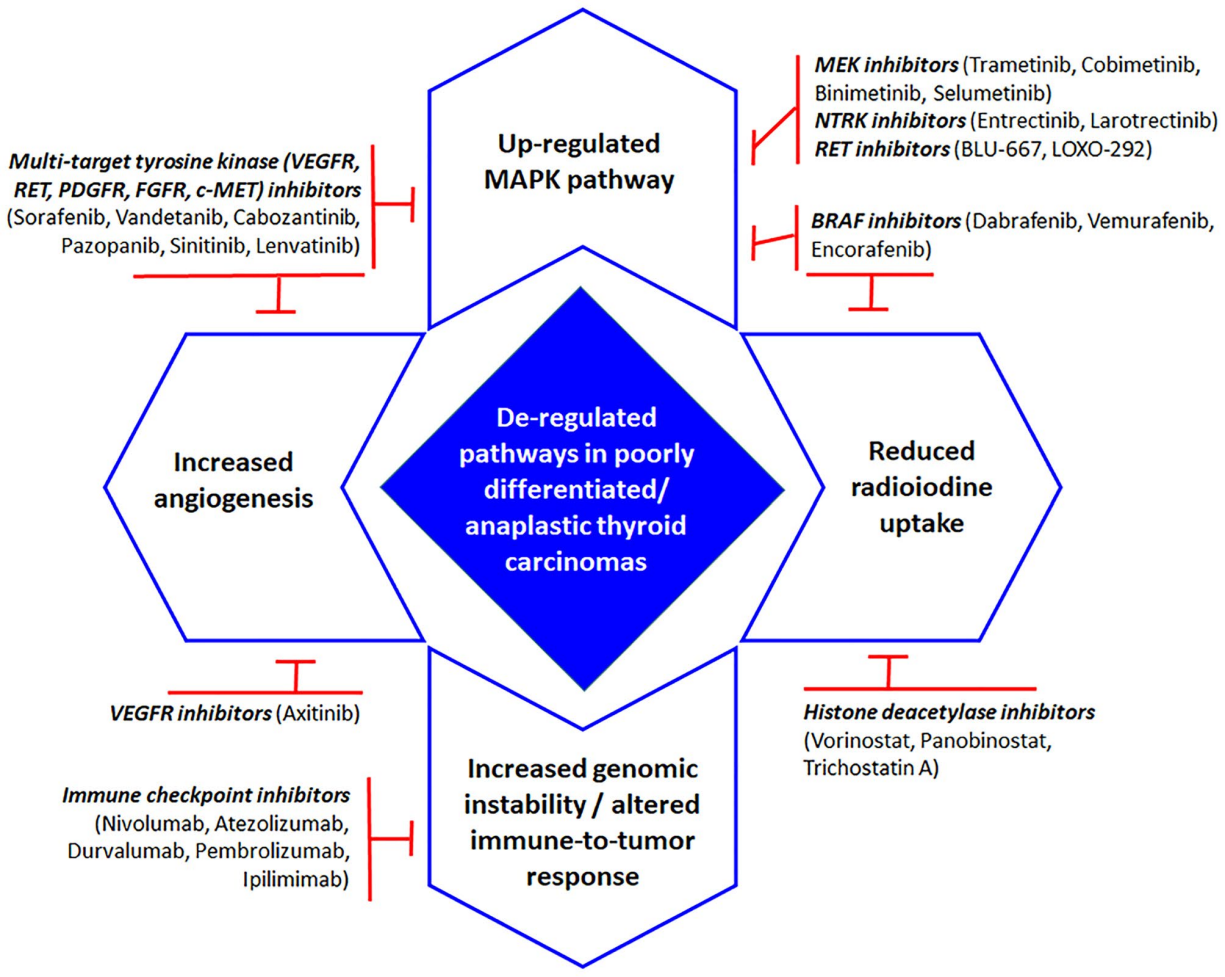
Schematic diagram of most relevant deregulated pathways and related drugs approved or under approval in poorly differentiated and anaplastic thyroid carcinomas (see ref. 82)

The need to identify genomic alterations that can be targeted by pathway-specific molecular drugs has been driving the attempt to clarify the molecular landscape of aggressive thyroid cancer types (Fig. 5) [82]. In fact, mutations in targetable pathways can be identified in approximately half of both poorly differentiated and anaplastic thyroid carcinomas and patients do benefit from molecularly targeted therapy when this is available [23]. Although the response to therapy is often independent of histologic typing, to clearly define the molecular background of poorly differentiated and anaplastic thyroid carcinomas is in principle essential to understand which are the tumors that should be prioritized for comprehensive genomic profiling.

Molecular alterations in thyroid cancer affecting genes/pathways that can be targeted by specific drugs in the current clinical scenario may be grouped in two main classes. The first class is represented by monoclonal antibodies whose role in treating thyroid cancer is, however, still uncertain. They are used against tyrosine kinase receptors or their ligands, mainly VEGF, to block angiogenesis [82], or more recently targeting immune checkpoint molecules especially in anaplastic thyroid cancer models [83]. Although immunotherapy represents an interesting alternative option also for aggressive thyroid cancer, very few clinical trials have been accomplished and very few studies exploring a way to overcome resistance have been performed [84]. Moreover, none of these agents is associated to molecular biomarkers indicative of response, if not in exploratory studies. The second group includes tyrosine kinase inhibitors that have been tested in the past 10 years for the treatment of advanced, progressive, radioiodine resistant thyroid tumors. Some of them have been approved for use in the clinical practice, such as sorafenib and lenvatinib. In other cancer types, such as colon and lung cancer, molecular testing is incorporated in the clinical practice to guide the selection of patients for individualized targeted therapy. However, in thyroid cancer clinical trials with different tyrosine kinase inhibitors have failed to identify molecular alterations predictive of response in both well differentiated and in poorly differentiated/anaplastic carcinomas. As an example, in the SELECT phase II study, the benefit in terms of progression-free survival of lenvatinib was maintained regardless of *BRAF* or *RAS* mutation status [85]. Thus, routine mutation profiling is not recommended at this time outside research settings.

In summary, the combination of genomic and epigenetic data shows that several molecular alterations affect druggable cellular pathways. These alterations represent indeed potential predictive biomarkers, but the clinical impact of molecular typing of poorly differentiated and anaplastic thyroid carcinomas at variance with other types of cancer is still an unmet need.
